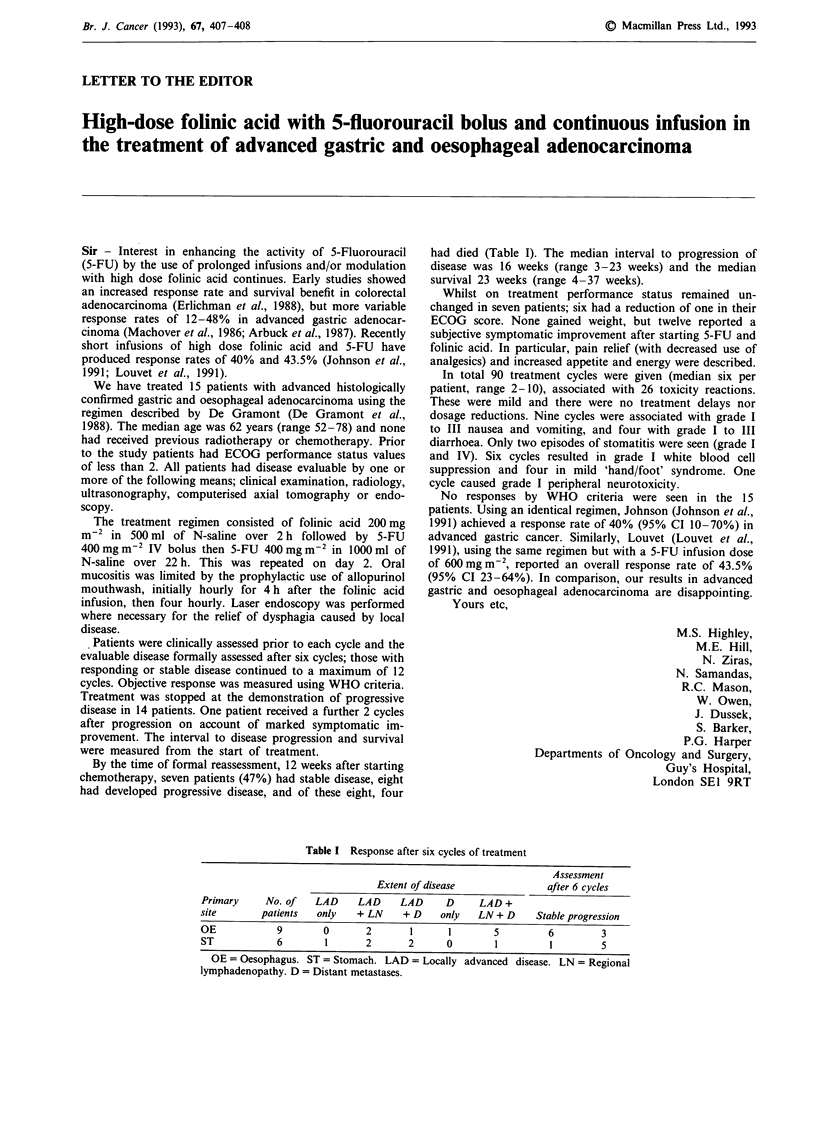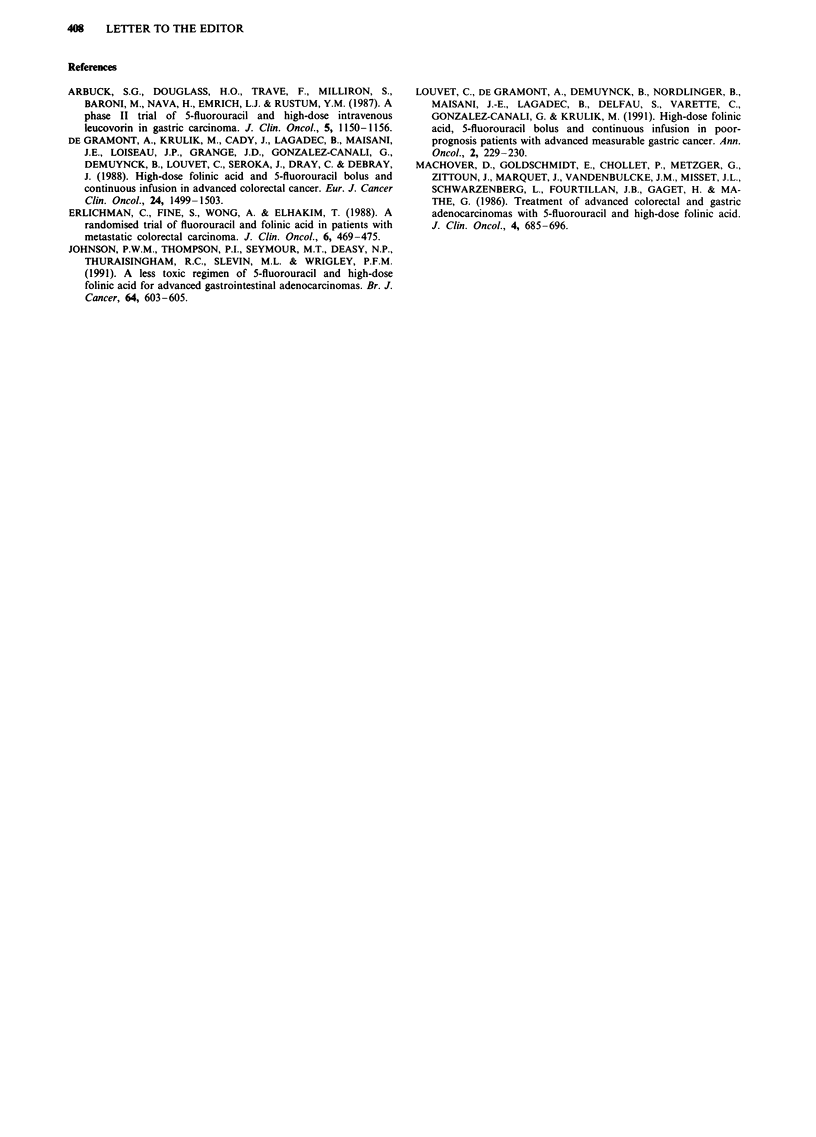# High-dose folinic acid with 5-fluorouracil bolus and continuous infusion in the treatment of advanced gastric and oesophageal adenocarcinoma.

**DOI:** 10.1038/bjc.1993.75

**Published:** 1993-02

**Authors:** M. S. Highley, M. E. Hill, N. Ziras, N. Samandas, R. C. Mason, W. Owen, J. Dussek, S. Barker, P. G. Harper


Br. J. Cancer (1993), 67, 407–408 © Macmillan Press Ltd., 1993

LETTER TO THE EDITOR

### High-dose folinic acid with 5-fluorouracil bolus and continuous infusion in the treatment of advanced gastric and oesophageal adenocarcinoma

**Sir** – Interest in enhancing the activity of 5-Fluorouracil (5-FU) by the use of prolonged infusions and/or modulation with high dose folinic acid continues. Early studies showed an increased response rate and survival benefit in colorectal adenocarcinoma (Erlichman *et al.*, 1988), but more variable response rates of 12–48% in advanced gastric adenocarcinoma (Machover *et al.*, 1986; Arbuck *et al.*, 1987). Recently short infusions of high dose folinic acid and 5-FU have produced response rates of 40% and 43.5% (Johnson *et al.*, 1991; Louvet *et al.*, 1991).

We have treated 15 patients with advanced histologically confirmed gastric and oesophageal adenocarcinoma using the regimen described by De Gramont (De Gramont *et al.*, 1988). The median age was 62 years (range 52–78) and none had received previous radiotherapy or chemotherapy. Prior to the study patients had ECOG performance status values of less than 2. All patients had disease evaluable by one or more of the following means; clinical examination, radiology, ultrasonography, computerised axial tomography or endoscopy.

The treatment regimen consisted of folinic acid 200 mg $m^{-2}$ in 500 ml of N-saline over 2 h followed by 5-FU 400 mg $m^{-2}$ IV bolus then 5-FU 400 mg $m^{-2}$ in 1000 ml of N-saline over 22 h. This was repeated on day 2. Oral mucositis was limited by the prophylactic use of allopurinol mouthwash, initially hourly for 4 h after the folinic acid infusion, then four hourly. Laser endoscopy was performed where necessary for the relief of dysphagia caused by local disease.

Patients were clinically assessed prior to each cycle and the evaluable disease formally assessed after six cycles; those with responding or stable disease continued to a maximum of 12 cycles. Objective response was measured using WHO criteria. Treatment was stopped at the demonstration of progressive disease in 14 patients. One patient received a further 2 cycles after progression on account of marked symptomatic improvement. The interval to disease progression and survival were measured from the start of treatment.

By the time of formal reassessment, 12 weeks after starting chemotherapy, seven patients (47%) had stable disease, eight had developed progressive disease, and of these eight, four had died (Table I). The median interval to progression of disease was 16 weeks (range 3–23 weeks) and the median survival 23 weeks (range 4–37 weeks).

Whilst on treatment performance status remained unchanged in seven patients; six had a reduction of one in their ECOG score. None gained weight, but twelve reported a subjective symptomatic improvement after starting 5-FU and folinic acid. In particular, pain relief (with decreased use of analgesics) and increased appetite and energy were described.

In total 90 treatment cycles were given (median six per patient, range 2–10), associated with 26 toxicity reactions. These were mild and there were no treatment delays nor dosage reductions. Nine cycles were associated with grade I to III nausea and vomiting, and four with grade I to III diarrhoea. Only two episodes of stomatitis were seen (grade I and IV). Six cycles resulted in grade I white blood cell suppression and four in mild 'hand/foot' syndrome. One cycle caused grade I peripheral neurotoxicity.

No responses by WHO criteria were seen in the 15 patients. Using an identical regimen, Johnson (Johnson *et al.*, 1991) achieved a response rate of 40% (95% CI 10–70%) in advanced gastric cancer. Similarly, Louvet (Louvet *et al.*, 1991), using the same regimen but with a 5-FU infusion dose of 600 mg $m^{-2}$, reported an overall response rate of 43.5% (95% CI 23–64%). In comparison, our results in advanced gastric and oesophageal adenocarcinoma are disappointing.

Yours etc,

M.S. Highley,
M.E. Hill,
N. Ziras,
N. Samandas,
R.C. Mason,
W. Owen,
J. Dussek,
S. Barker,
P.G. Harper
Departments of Oncology and Surgery,
Guy's Hospital,
London SE1 9RT

**Table I** Response after six cycles of treatment

| | | *Extent of disease* | | | | | *Assessment after 6 cycles* | |
|---|---|---|---|---|---|---|---|---|
| *Primary site* | *No. of patients* | *LAD only* | *LAD + LN* | *LAD + D* | *D only* | *LAD + LN + D* | *Stable* | *progression* |
| OE | 9 | 0 | 2 | 1 | 1 | 5 | 6 | 3 |
| ST | 6 | 1 | 2 | 2 | 0 | 1 | 1 | 5 |

OE = Oesophagus. ST = Stomach. LAD = Locally advanced disease. LN = Regional lymphadenopathy. D = Distant metastases.

**References**

ARBUCK, S.G., DOUGLASS, H.O., TRAVE, F., MILLIRON, S., BARONI, M., NAVA, H., EMRICH, L.J. & RUSTUM, Y.M. (1987). A phase II trial of 5-fluorouracil and high-dose intravenous leucovorin in gastric carcinoma. *J. Clin. Oncol.*, **5**, 1150–1156.

DE GRAMONT, A., KRULIK, M., CADY, J., LAGADEC, B., MAISANI, J.E., LOISEAU, J.P., GRANGE, J.D., GONZALEZ-CANALI, G., DEMUYNCK, B., LOUVET, C., SEROKA, J., DRAY, C. & DEBRAY, J. (1988). High-dose folinic acid and 5-fluorouracil bolus and continuous infusion in advanced colorectal cancer. *Eur. J. Cancer Clin. Oncol.*, **24**, 1499–1503.

ERLICHMAN, C., FINE, S., WONG, A. & ELHAKIM, T. (1988). A randomised trial of fluorouracil and folinic acid in patients with metastatic colorectal carcinoma. *J. Clin. Oncol.*, **6**, 469–475.

JOHNSON, P.W.M., THOMPSON, P.I., SEYMOUR, M.T., DEASY, N.P., THURAISINGHAM, R.C., SLEVIN, M.L. & WRIGLEY, P.F.M. (1991). A less toxic regimen of 5-fluorouracil and high-dose folinic acid for advanced gastrointestinal adenocarcinomas. *Br. J. Cancer*, **64**, 603–605.

LOUVET, C., DE GRAMONT, A., DEMUYNCK, B., NORDLINGER, B., MAISANI, J.-E., LAGADEC, B., DELFAU, S., VARETTE, C., GONZALEZ-CANALI, G. & KRULIK, M. (1991). High-dose folinic acid, 5-fluorouracil bolus and continuous infusion in poor-prognosis patients with advanced measurable gastric cancer. *Ann. Oncol.*, **2**, 229–230.

MACHOVER, D., GOLDSCHMIDT, E., CHOLLET, P., METZGER, G., ZITTOUN, J., MARQUET, J., VANDENBULCKE, J.M., MISSET, J.L., SCHWARZENBERG, L., FOURTILLAN, J.B., GAGET, H. & MATHE, G. (1986). Treatment of advanced colorectal and gastric adenocarcinomas with 5-fluorouracil and high-dose folinic acid. *J. Clin. Oncol.*, **4**, 685–696.